# Sphingomyelin-Based Nanosystems (SNs) for the Development of Anticancer miRNA Therapeutics

**DOI:** 10.3390/pharmaceutics12020189

**Published:** 2020-02-22

**Authors:** Surasa Nagachinta, Belen Lopez Bouzo, Abi Judit Vazquez-Rios, Rafael Lopez, Maria de la Fuente

**Affiliations:** 1 Nano-Oncology Unit, Health Research Institute of Santiago de Compostela (IDIS), SERGAS, 15706 Santiago de Compostela, Spain; surasa.nagachinta@gmail.com (S.N.); lopezbouzobelen@gmail.com (B.L.B.);; 2 Translational Medical Oncology group (ONCOMET), Health Research Institute of Santiago de Compostela (IDIS), SERGAS, 15706 Santiago de Compostela, Spain; 3 Center for Research in Molecular Medicine and Chronic Diseases (CIMUS), Department of Pharmacy and Pharmaceutical Technology, School of Pharmacy, University of Santiago de Compostela, Campus Vida, 15706 Santiago, Spain; 4 Centro de Investigación Biomédica en Red Cáncer (CIBERONC), 28029 Madrid, Spain

**Keywords:** sphingomyelin, nanomedicine, gene therapy, oncosuppressor miRNAs, colorectal cancer

## Abstract

Gene replacement therapy with oncosuppressor microRNAs (miRNAs) is a promising alternative to interfere with cancer progression. However, miRNAs are highly inefficient in a biological environment, hampering a successful translation to clinics. Nanotechnology can tackle this drawback by providing delivery systems able to efficiently deliver them to cancer cells. Thus, the objective of this work was to develop biocompatible nanosystems based on sphingomyelin (SM) for the intracellular delivery of miRNAs to colorectal cancer cells. We pursued two different approaches to select the most appropriate composition for miRNA delivery. On the one hand, we prepared sphingomyelin-based nanosystems (SNs) that incorporate the cationic lipid stearylamine (ST) to support the association of miRNA by the establishment of electrostatic interactions (SNs–ST). On the other hand, the cationic surfactant (DOTAP) was used to preform lipidic complexes with miRNA (Lpx), which were further encapsulated into SNs (SNs-Lpx). Restitution of miRNA145 levels after transfection with SNs-Lpx was related to the strongest anticancer effect in terms of tumor proliferation, colony forming, and migration capacity assays. Altogether, our results suggest that SNs have the potential for miRNA delivery to develop innovative anticancer therapies.

## 1. Introduction

Nowadays, cancer is one of the leading causes of death [1]. Cancer treatments can still be improved, making use of novel technologies and biotechnological drugs. Advances in nanotechnology have shown a prompt impact in numerous applications in cancer, since nano-scaled carriers have the potential for incorporating various kinds of molecules into their architecture, including anticancer drugs and contrast agents, as well as macromolecules, such as proteins, peptides, or oligonucleotides [2,3]. Indeed, several nanoformulations, mainly liposomes, have been successfully translated to the clinical practice [4,5].

RNA-based therapeutics, such as small interfering RNA (siRNA) and microRNA (miRNA), provide a promising approach to treat cancer by targeting specific proteins involved in the mechanism of proliferation, invasion, antiapoptosis, drug resistance, and metastasis [6,7]. miRNAs are small (17 to 25 nucleotides) non-coding RNA molecules, which specifically interact with target messenger RNA (mRNAs), resulting in inhibited translation or mRNA cleavage and degradation [8]. It is well known that various miRNAs, for instance, miRNA-10a, miRNA-34b/c, miRNA-137, miRNA-143, and miRNA-145, are downregulated in colorectal cancer cells compared to healthy tissues, and modulation of the corresponding gene expressions is gaining interest for the development of anticancer therapeutics [9,10,11,12,13]. However, the major barriers of miRNA delivery are (i) poor systemic stability, (ii) rapid clearance, (iii) degradation by nucleases, (iv) risk of systemic toxicity, (v) elimination by phagocytic immune cells, and (vi) lack of efficient delivery to targeted cells to achieve the desired therapeutic outcome [14]. For overcoming these constraints, different delivery systems have been proposed to date, such as PLGA/PEI/miRNA/HA nanoparticles, protamine nanoparticles, and magnetic nanoparticles [15,16,17].

miRNA-nanotherapeutics might represent a promising alternative for the development of cancer therapeutics. For the design of nanostructures, it is possible to use organic biodegradable materials that do not accumulate in the body and do not cause toxicity. Lipidic sphingomyelin-based nanosystems (SNs), of which the main components are sphingomyelin, present in cell membranes, and vitamin E, widely used in different formulations that are of clinical use, are promising carriers due to their high biocompatibility and versatility for the association of a variety of drugs [18,19]. In this study, we aimed to optimize the nanoformulations based on SNs, for their specific application in cancer gene therapy, to mediate an efficient association and delivery of oncosuppressor miRNA-145 and treat colorectal cancer. For this, two different strategies were pursued. Firstly, the cationic lipid stearylamine was incorporated into the nanoparticles, in order to obtain cationic SNs and favor the association of miRNA mimics (SNs-ST). Secondly, we proposed the encapsulation of preformed DOTAP-miRNA lipid complexes (Lpx) into SNs (SNs-Lpx) to provide additional protection for the associated miRNA mimics, following a similar approach to that described for plasmid DNA (pDNA), complexed via hydrophobic ion pairing utilizing surfactants and further incorporated into self-nanoemulsifying drug delivery systems [20,21]. Intracellular delivery, cell transfection, and functional in vitro assays were accomplished to determine the potential of the proposed formulations to develop new oncosuppressor therapies for colorectal cancer.

## 2. Materials and Methods

### 2.1. Materials

Vitamin E (dl-α-tocopherol) was obtained from Merck (Darmstadt, Germany). Sphingomyelin (SM) and *N*-[1-(2,3-Dioleoyloxy)propyl]-*N*,*N*,*N*-trimethylammonium methylsulfate (DOTAP) were acquired from Lipoid (Ludwigshafen, Germany). Stearylamine (ST), agarose, heparin, Mowiol^®^ 4-88, Dulbecco’s Modified Eagle’s Medium (DMEM), and Dulbecco’s Phosphate Buffered Saline (PBS) were purchased from Sigma Aldrich (St.Louis, MO, USA). PEG_12_-C_18_ was supplied by Creative PEGworks (Chapel Hill, NC, USA). *N*-[11-(dipyrrometheneboron difluoride)undecanoyl]-d-erythro sphingosylphosphoryl choline (SM-TopFluor^®^) was purchased from Avanti Polar Lipids (Alabaster, AL, USA). RNAs were synthesized by Eurofins Genomics (Ebersberg, Germany) (miRNA145: 5′ GUCCAGUUUUCCCAGGAAUCCCU 3′; miRNA scramble (miRNAscr): 5′ UUCUCCGAACGUUGUCACGUUU 3′; Cy5-modified miRNA145 (miRNA-Cy5): 5’ Cy5- GUCCAGUUUUCCCAGGAAUCCCU 3′). SYBR^®^ Gold Nucleic Acid Gel Stain, RNAse free water, DNA ladder, penicillin, and streptomycin were obtained from Invitrogen (Madrid, Spain). A microRNA Purification Kit was acquired from Norgen Biotek Corporation (Zaragoza, Spain). qScriptTM microRNA cDNA Synthesis kit, PerfeCta^®^ Universal PCR Primer, and PerfeCta^®^ SYBR^®^ Green SuperMix, Low RoxTM were bought from Quantabio, VWR International Eurolab (Barcelona, Spain). Universal primers: hsa-miR145-5p (5′-CGCGCGTTCCAGTTTTCCCAGG-3′), universal reverse PCR primer (5′-GTGCAGGGTCCGAGGT-3′) and the housekeeping small RNA control primer RNU6 (5′CTCGCTTCGGCAGCACA3′, 5′AACGCTTCACGAATTTGCGT3′) were purchased from Fisher Scientific (Madrid, Spain). SW480 cells were obtained from ATCC (ATCC^®^ CCL-228™). Ultrapure Mili-Q water was used all throughout the experiments. Ethanol was of analytical grade and supplied from Thermo Fisher Scientific (Madrid, Spain). All other chemicals used were of reagent grade.

### 2.2. Preparation and Characterization of SNs

SNs were formulated with vitamin E (VitE), sphingomyelin (SM), and PEG_12_-C_18_ (PEG) in a ratio of VitE:SM:PEG (10:1:0.1 *w*/*w*), by ethanol injection method. In brief, the components were dissolved in a final volume of 100 μL in ethanol and injected into ultrapure water (1 mL) under magnetic stirring (total lipid concentration 5.05 mg/mL). SNs were isolated from non-interacted compounds by ultracentrifugation (Beckman Coulter, Brea, CA, USA) at 35,000 rpm for 1 h at 15 °C in a 70.1 Ti rotor, obtaining a cream consisting of the isolated SNs on top of the aqueous phase.

### 2.3. Preparation and Characterization of Cationic Stearylamine SNs (SNs-ST) and Association of miRNA (miR)

Cationic SNs were prepared upon addition of the cationic lipid, stearylamine (ST) to the organic phase at a ratio of VitE:SM:PEG:ST 10:1:0.1:1 (*w*/*w*). SNs-ST were fully characterized. miRNA was subsequently associated to SNs-ST by the establishment of electrostatic interactions performing a simple incubation. A total of 10 μL of miRNA (10 μg) were incubated with 90 μL of SNs-ST at different miRNA:ST mass ratios (1:10, 1:7.5, 1:5, 1:2.5, and 1:1) to obtain theoretical loadings ranging from 0.8% to 7.6% (*w*/*w*) with respect to the total mass of the nanosystems. The association of miRNA was analyzed by agarose gel electrophoresis (1% *w/v* in Tris-Acetate-EDTA (TAE) Buffer). Briefly, 0.5 μg of nucleic acids labeled with SYBR^®^ Gold, either in solution, associated to the nanoparticles, or after displacement with an excess of heparin (25-fold heparin with respect to the amount of miRNA for 2 h at 37 °C) were loaded into each well. Gel electrophoresis was run at 100 V, 40 min in a Sub-Cell GT cell 96/192 (Bio-Rad Laboratories Ltd., Deeside, England). Gel images were obtained with a Molecular Imager^®^ Gel DocTM XR System (UV light 302 nm; Bio-Rad, Madrid, Spain).

### 2.4. Preparation and Characterization of Lipid Complexes of miRNA with Cationic Lipids

Preparation of lipid complexes (Lpx) was attempted with miRNA and cationic lipids (ST or DOTAP). A total of 10 μg of miRNA were incubated with cationic lipid at different miRNA: cationic lipid mass ratios (1:1, 1:5, 1:10, 1:15, and 1:20) in a total solution of 500 μL (H_2_O 450 μL and EtOH 50 μL). They were characterized by their physicochemical properties.

### 2.5. Loading of Lpx into SNs (SNs-Lpx)

miRNA:DOTAP Lpx in a ratio of 1:15 were lyophilized using a VirTis GenesisTM 25 EL (Warminster, PA, USA). Lyophilization steps contained thermal treatment, freezing, primary drying, and secondary drying. It was performed at a temperature ranging from −40 °C to +20 °C, applying a progressive vacuum from 200 mTorr to 20 mTorr. Lyophilized Lpx were characterized by DLS. A total of 50 μL of resuspended Lpx in ethanol were diluted in 450 μL of ultrapure water and subsequently analyzed. For preparation of SNs-Lpx, lyophilized Lpx were suspended in 100 μL of the organic phase (containing VitE, SM, and PEG_12_-C_18_, in a ratio of 10:1:0.1 (*w*/*w*) and immediately injected to water (1 mL). The association efficiency of miRNA was studied by gel agarose gel electrophoresis, following the same protocol as described in the previous Section 2.3. In this particular case, broken SNs-Lpx (by mixing the formulation with ethanol at 1:5 *v*/*v*) were additionally incubated with a 25-fold heparin with respect to the amount of miRNA, for 2 h at 37 °C.

### 2.6. Characterization of Nanosystems (Size and Zeta-Potential Measurements, Transmission Electron Microscopic (TEM), and Nanoparticle Tracking Analysis (NTA))

Nanoparticles were analyzed for their hydrodynamic size and polydispersity index (PDI) by dynamic light scattering (DLS) using a Zetasizer Nano ZS (Malvern Instruments, Worcestershire, UK) equipped with a standard λ = 633 nm laser as the incident beam. They were diluted in ultrapure water and loaded into a disposable solvent resistant microcuvette (ZEN0040). The obtained data were analyzed based on a cumulative analysis method for determination of mean hydrodynamic diameter and PDI. ζ–potential, a parameter indicative of the surface charge of the nanocarriers, was measured by Laser Doppler Anemometry (LDA) in the same equipment, using a dip-cell (DTS 1060). Each analysis was performed in triplicate at 25 °C.

The morphology of SNs was investigated using TEM (CM12 Philips; Eindhoven, the Netherlands). A total of 10 μL of diluted nanosystems (1/10 in Milli-Q water) were placed on 400-mesh copper grids, incubated for 3 min at room temperature, and stained with 10 μL of 2% phosphotungstic acid solution for 1 min. Excessive solution of phosphotungstic acid was removed with filter paper. The grids were washed five times in water droplets for 1 min (each time) and dried overnight under vacuum.

NTA measurements were performed with a NanoSight NS300 (Malvern Instruments, Worcestershire, UK), equipped with a sample chamber with a 488-nm laser. Samples were diluted 1/1000 in Milli-Q water and then injected in the sample chamber with sterile syringes (Omnifix^®^-F 1ml, Melsungen, Germany). All measurements were performed at room temperature for 60 s in triplicate. The mean particle size and standard deviation values obtained by the NTA software correspond to the arithmetic values calculated with the sizes of all the particles analyzed by the software.

### 2.7. In Vitro Cell Uptake

SW480 cells were cultured in DMEM supplemented with 10% (*v*/*v*) of FBS and 1% (*v*/*v*) of penicillin-streptomycin in a humidified atmosphere with 5% CO_2_ at 37 °C. All in vitro studies were performed in this setting, unless otherwise stated.

To determine the working conditions, the cytotoxicity of SNs-ST and SNs-Lpx was assessed by the 3-(4,5-dimethylthiazol-2-yl)-2,5-diphenyltetrazolium bromide (MTT, Thermo Fisher Scientific) assay. Briefly, cells were seeded at a density of 10^4^ cells/well in a 96-well plate containing 100 μL of fresh culture medium and incubated overnight to allow cell attachment for subsequent study. Then, cells were cultured in the presence of different concentrations for 24 h at 37 °C. After the incubation, MTT (5 mg/mL) was added to medium and further incubated for 4 h, then 100 μL DMSO was added to dissolve the formazan crystals formed in the live cells for 10 min at 37 °C. The absorbance at 570 nm was recorded using a spectrometer.

Confocal laser-scanning microscope (TCS SP5, Leica Microsystems GmbH, Heidelberg, Germany) was used to observe cell uptake of fluorescent nanoparticles. Twenty-four hours before the experiment, SW480 cells (1 × 10^5^ cells/well) were seeded onto 12 mm diameter glass coverslips in a 24-well plate in 0.5 mL of supplemented cell culture medium. SNs-ST and SNs-Lpx were labeled with SM-TopFluor^®^ (0.2 ng/μL) and miRNA-Cy5 (0.2 ng/μL) to allow direct observation of the molecule of interest. After addition of 20 μL of the formulation to the cells, they were incubated for 4 h at 37 °C in the cell incubator. After that, cells were exhaustively washed with PBS and then fixed with paraformaldehyde (PFA; 4% *v/v* in PBS) in the dark at room temperature for 15 min. Cells were rinsed again with PBS, and cell nuclei were then counterstained with Hoechst 33342 (1:1000 in PBS) for 5 min. They were washed again with PBS. Lastly, 8 μL of Mowiol^®^ 4-88 were used for mounting samples on coverslips. Preparations were conserved in the dark at −20 °C. The confocal laser scanning microscopic images were obtained with a 63× oil immersion objective for Hoechst 33342 (blue), SM-TopFluor^®^ (green), and miRNA-Cy5 (red), respectively (scanning speed 600 Hz, with an image resolution of 1024 × 1024 pixels). The co-localization ratio of miRNA-Cy5 and SM-TopFluor^®^ was determined by LAS AF software (Barcelona, Spain).

Studies were also accomplished by Fluorescence-Activated Cell Sorting (FACScan flow cytometer, BD biosciences, San Jose, CA, USA). For this experiment, the cells were transfected with the same amount of fluorescent formulations as mentioned in the confocal study. They were incubated for 4 h at 37 °C in the cell incubator and washed with PBS. Then, the cells were trypsinized and resuspended in 0.5 mL of PFA (approx. 1 × 10^5^ cells/mL) prior to analysis. The results were analyzed using Flowjo 8.7 (Ashland, OR, USA).

### 2.8. Transfection Efficiency

SNs-ST, Lpx, and SNs-Lpx were evaluated for their transfection efficacy in SW480 human colorectal cancer cells. All types of nanosystems were formulated with miRNA145 (miR145), and with a scrambled sequence (miRScr). SW480 cells were seeded in 6-well plates (5 × 10^5^ cells/well) and incubated in completed DMEM for 24 h. Formulations were then added (SNs-ST (miR145), SNs-ST (miRScr), Lpx (miR145), Lpx (miRScr), SNs-Lpx (miR145), and SNs-Lpx (miRScr), being the dose 2 μg of miRNA in a final volume of 2 mL of fresh cell culture medium without supplements. The nanocarriers were removed after 4 h of incubation, cells washed, and fresh completed medium added (2 mL). The transfection efficiency was determined 72 h post-transfection by quantitative real-time PCR (qRT-PCR) (Stratagene Mx 3000, Agilent Technologies). According to the manufacturer’s protocol of Norgen Biotek Corporation, microRNA Purification Kit (Thorold, ON, Canada), the total miRNA was extracted from SW480 cells. miRNA concentration and purity were evaluated with UV spectrophotometry (Nanodrop, Spectrophotometer ND-100, Thermo Scientific). Extracted RNA samples were measured and diluted to have the same amount of RNA (120 ng). Next, the reverse RNA transcription to cDNA was carried out using qScript™ microRNA cDNA Synthesis Kits (Quanta Biosciences). The qRT-PCR was performed using PerfeCta^®^ MicroRNA Assays (Quanta Biosciences), with a primer for miRNA 145 (has-miR145-5p). Small nuclear RNA, RNU-6 was employed as an endogenous housekeeping gene to normalize the miRNA amount. PCR cycle consisted of activation at 95 °C (2 min), denaturation at 95 °C (5 s), and annealing at 60 °C (30 s) for 40 cycles. Quantitative data were analyzed by utilizing an AriaMx Real-time PCR System (Agilent Technologies). The relative miRNA145 expression level was calculated based on the comparative 2^−ΔΔCT^ method in relation to RNU6 and normalized to that obtained from non-treated cells.

### 2.9. Functional Assays

For cell proliferation assay, transfected cells were seeded at 2 × 10^5^ cells/well onto 24-well plate and harvested at 72 h post-transfection. Cells were then counted manually in a Neubauer-improved cell counting chamber, depth 0.100 mm, 0.0025mm^2^ (Marienfeld, Germany). With respect to the cell migration assay, after transfection, cells were trypsinized and counted. Then, 2 × 10^5^ cells/well were planted onto a 24-well plate, and artificial wounds were created on the confluent cell monolayer using a 200 μL pipette tip. Wounded cells were gently washed PBS for 3 times to remove the detached cells and cultures in 5% CO_2_ at 37 °C. The wound closure was observed and photographed at time point 0, 24, 48, 72, and 96 h under microscope (Leica type 090-135.00, Wetzlar, Germany). The wound closure area was calculated by analyzing the microscopy images with the software ImageJ (1.48d; National Institutes of Health, Bethesda, MD, USA). For colony-forming assay, transfected cells were seeded at a density of 200 cells/well in 12-well plates and cultured at 37 °C for 2 weeks. Cells were stained with MTT solution (5 mg/mL) and photographed. The images were analyzed using ImageJ software.

### 2.10. Statistical Analysis

Differences were statistically determined by one-way ANOVA followed by Tukey’s method. All statistical analysis was performed using GraphPad Prism (Version 6.0 software) (GraphPad Software, San Diego, CA, USA). A *p* value < 0.05 was considered to be significant.

## 3. Results and Discussion

### 3.1. Development and Characterizations of miRNA-Loaded SNs

**Figure 1:**
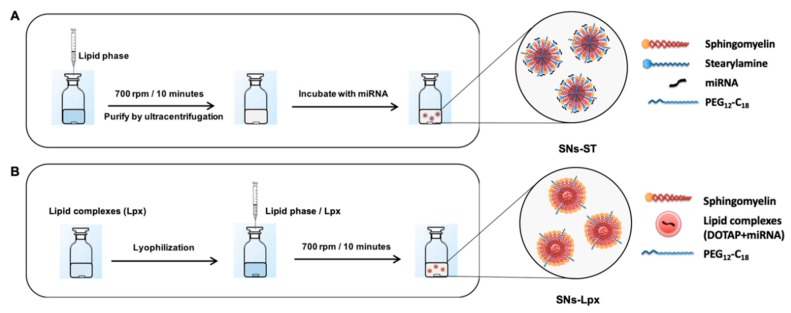
Preparation and scheme-illustrated components of (**A**) sphingomyelin-based nanosystems (SNs)-stearylamine (ST) and (**B**) SNs-Lipid complexes (Lpx).

Lipid-based nanoparticles have been widely used in gene delivery. Incorporation of cationic lipids, such as DOTMA, DOTAP, ST, and DC-Chol (3β-[*N*-(*N*’,*N*’-dimethylaminoethane)-carbamoyl] cholesterol), allowed an efficient association of nucleic acids onto the particle surface [22,23,24,25]. On the other hand, emulsions composed by biocompatible and biodegradable lipids have been widely reported in clinical formulations, thus ensuring a recognized safety profile [26]. In this study, we optimized the composition of SNs, previously reported by our group [18,19], to extend their use for gene therapy applications in cancer, and more concretely for the delivery of oncosuppressor miRNAs for the treatment of colorectal cancer. SNs were prepared following the ethanol injection method, a simple and fast procedure that avoids drastic pH changes and high-energies while rendering stable and reproducible formulations. At this point, we selected two strategies for an efficient miRNA delivery. Our first strategy (Figure 1A) was to adsorb the nucleic acids onto the surface of preformed cationic SNs prepared with stearylamine (SNs-ST). While the second approach was to prepare lipid complexes (Lpx) with cationic lipids, for subsequent encapsulation into SNs (SNs-Lpx) (Figure 1B).

#### 3.1.1. Development and Characterization of SNs-ST

Cationic SNs were prepared by including an additional component to the formulation, the cationic lipid stearylamine (ST), with the reported capacity to mediate an efficient association of nucleic acids through the establishment of electrostatic interactions since it presents a positively charged primary ammonium group over a wide range of pH [27,28]. Additionally, ST has already been described for the preparation of cationic nanoemulsions composed of pDNA for delivery of nucleic acids [29]. As shown in Table 1, SNs prepared with increasing amounts of ST rendered cationic formulations (zeta potential ranging from +40 to +51 mV) with a similar nanoparticle size of around 100 nm in all cases. In general, all SNs-ST formulations present a slightly smaller size than blank SNs (mean average size of 131 ± 8 nm), and an inversion on the zeta potential, properties that evidence the efficient incorporation of ST to the formulation. Nanoparticle tracking analysis (NTA) analysis of SNs correlates with DLS analysis, confirming the size and the mono-disperse size distribution, and giving a concentration of 4.2 × 10^12^ ± 2.1 × 10^10^ particles/mL (Figure S1). SNs-ST prepared with the highest amounts of ST (ratios 1.5 and 2) showed a higher polydispersity index (0.3). Based on the described physicochemical properties, formulations prepared with an intermediate amount of ST (ratio of 1) were selected for further studies.

After having selected a formulation with optimal physicochemical properties for gene therapy (it is known that surface properties greatly influence the capacity of cationic nanosystems to associate nucleic acids [30,31], and that size affects their cellular entry, transfection efficiency, and in vivo biodistribution [32,33]), association of miRNA to SNs-ST was attempted. A constant amount of miRNA (10 μg) was added over decreasing amounts of SNs-ST, under magnetic stirring. The assayed miRNA:ST ratios ranged from 1:10 to 1:1 (*w*/*w*), which translated into theoretical loadings from 0.8 to 7.6% (*w*/*w*) with respect to the total mass of the formulation. The physicochemical properties of the loaded formulations are shown in Table 2. As it can be observed, decreasing the proportion of SNs-ST, and therefore of cationic groups, mainly affects the surface properties of the nanosystems. While proportions of miRNA:ST 1:10 rendered cationic nanosystems, an inversion on the zeta potential was reported for formulations prepared with the two lower proportions of ST (miRNA:ST 1:1 and 1:2.5). Sizes ranging from 150 to 180nm, and low polydispersity indexes, were reported in all cases except for the ratios miRNA:ST 1:5 and 1:7.5, for which a massive precipitation was observed, most probably due to the neutralization of the surface charge, as reported for other types of RNA/DNA-loaded nanosystems in which preparation relies on the establishment of electrostatic interactions [34,35].

The association of the miRNA onto the surface of SNs-ST was confirmed by a gel electrophoresis assay (Figure S2). Results allowed us to select a specific miRNA:ST mass ratio of 1:10 for the next experiments (no free miRNA was observed for this specific composition, Figure S2). Characterization was completed after analysis by transmission electron microscopy (TEM), NTA, and DLS (Figure S3). TEM images showed a clear vesicle structure (spherical morphology) of SNs-ST and SNs-ST (miR). The releasing of miRNA from SNs-ST was determined by heparin displacement assay, a widely accepted method for characterization of gene nanocarriers, polyplexes, and lipoplexes [36]. From Figure S2, a band corresponding to free unbound miRNA after the incubation of SNs-ST (miR) with heparin can be observed. This indicated that the negatively charged molecule miRNA was at least partially displaced with high concentrations of the negatively charged heparin, indicating that an additional layer of protection, as for example, reported for protamine nanocapsules [17], could greatly improve their biological performance. A different strategy is the one reported in this work (see next section), with the encapsulation of the nucleic acids inside SNs previous complexation with cationic lipids (SNs-Lpx)

#### 3.1.2. Development and Characterization of SNs-Lpx

Pre-condensation of DNA with polycations has been described to be a favorable method to increase in vitro and in vivo transfection efficiency of lipid nanoparticles. This enhancement is due to the formation of compacted complexes supporting encapsulation of nucleic acids and additional protection against enzymatic degradation [37,38]. Moreover, the complexation of a model plasmid with different cationic lipids via hydrophobic ion pairing (HIP) has been described to reduce hydrophilicity of pDNA, enabling its incorporation into a lipophilic carrier, and enhancing its stability in biological environments [39]. Similarly, modification by HIP has been described to increase the lipophilicity of peptides, allowing further association to lipid nanosystems [40]. In our case, we followed this approach for complexation of miRNAs and associated them with SNs. Within this study, HIP was established among the amino group of the cationic lipid and the negatively charged phosphate group of the RNA backbone.

Lpx were prepared at first instance with ST (at various miRNA:ST ratios), but after characterization of the resulting Lpx (ST) formulations, poor results in terms of size and polydispersity index let us disregarding these compositions (Table S1). DOTAP was then explored as an alternative cationic lipid since it is available as GMP material for clinical studies, and holds a positive charge at a pharmaceutically relevant pH, which is appropriated for gene carriers [41]. In general, most of the Lpx (DOTAP) had a broad PDI and were, therefore, disregarded (Table S1). Due to the smallest size of the miRNA:DOTAP 1:15 ratio, around 80 nm with a PDI 0.3, this specific condition was selected for the preparation of Lpx to be further encapsulated into SNs. Lpx were freeze-dried and characterized after resuspension in ethanol to show that they preserve their properties during the procedure (size 72 ± 17 nm, PDI 0.3).

**Figure 2:**
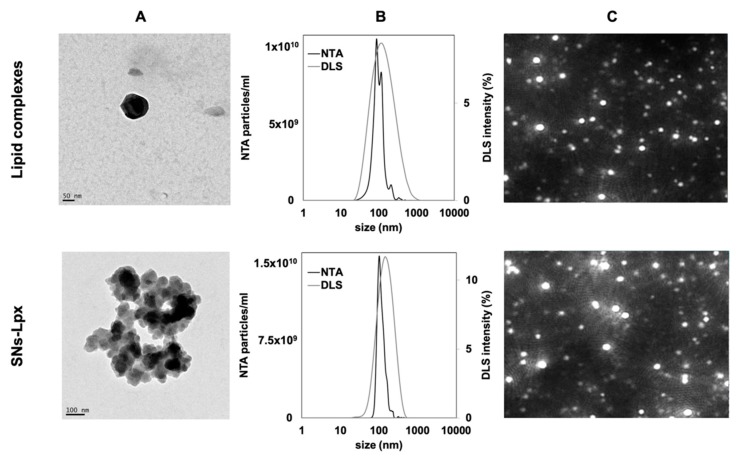
Characterization of lipid complexes and SNs-Lpx. (**A**) Transmission electron microscopy (TEM) images. (**B**) Size distribution graph measured from nanoparticle tracking analysis (NTA) and dynamic light scattering (DLS), and (**C**) video frame acquired by NTA.

Preparation of SNs-Lpx involved resuspension of freeze-dried Lpx into the ethanol phase containing the lipid mixture (VitE:SM:PEG 10:1:0.1), prior injection into ultrapure water (see Figure 1B). SNs-Lpx nanosystems exhibited a mean particle size of 124 ± 9 nm, a PDI of 0.2, and a positive ζ-potential of +37 ± 3. SNs-Lpx were additionally characterized by TEM and NTA. While TEM offers a direct visualization of the particles, NTA provides a more dynamic measurement by identifying and tracking individual particles and adds information related to the accurate concentration of the particles [42,43]. TEM images revealed the spherical morphology of SNs-Lpx (Figure 2). DLS and NTA data proved that SNs-Lpx nanosystems were homogeneous in size and monodisperse. Altogether, results obtained by the different complementary techniques show a single population of homogenous nanoparticles, indicative of the successful inclusion of the Lpx within SNs.

Finally, a gel electrophoresis assay additionally proved that miRNA was efficiently incorporated into SNs-Lpx (Figure S4), and that the inclusion of the Lpx into SNs offers an additional layer of protection, avoiding its premature release in contact with anions. After a heparin displacement experiment, free (displaced) miRNA was only observed for Lpx, and when SNs-Lpx were previously treated with alcohol, breaking the emulsions, but not in the case of the intact formulation, as the complex was not accessible for heparin.

### 3.2. In Vitro Cell Uptake

Apart from the characterization of the nanoformulations, good interaction and internalization with target cells are required for mediating an anticancer effect. To determine the working conditions, first, experiments were carried out to determine the toxicity of SNs-ST and SNs-Lpx in the targeted SW480 cell line, as shown in Figure S5. Importantly, these studies confirmed the biocompatibility of the formulations, as toxic effects were not observed after incubation at concentrations up to 0.5 mg/mL for 24 h, in line with previous reports performed with other types of nanocarriers considered to be safe, as for example, cationic nanoemulsions [42].

**Figure 3:**
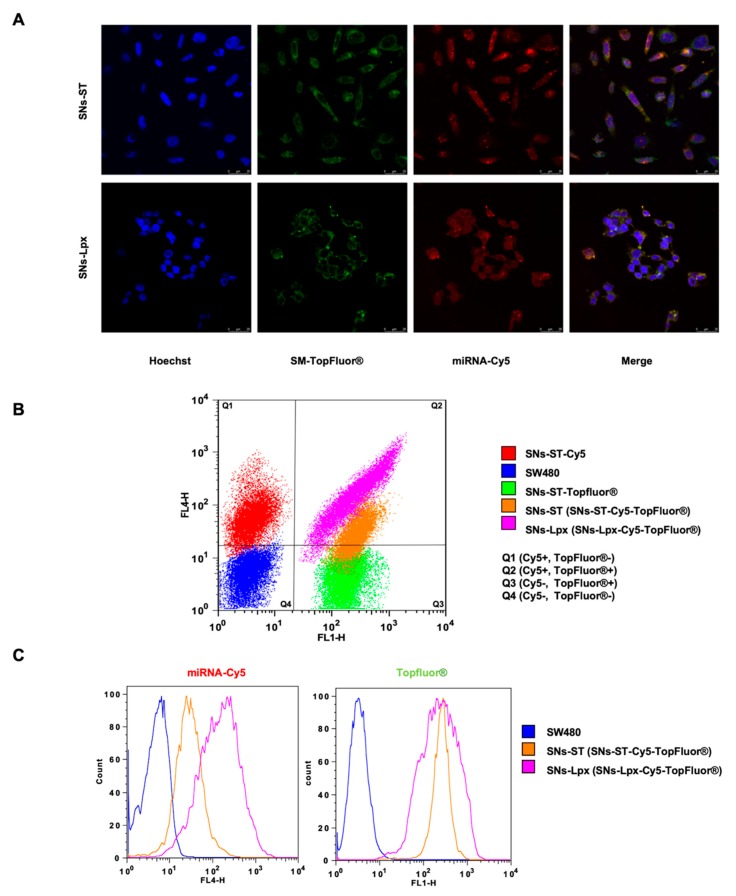
(**A**) Internalization of SNs-ST and SNs-Lpx in SW480. Maximal projection of confocal images upon incubation of nanosystems labeled with Sphingomyelin TopFluor^®^ (green), and miRNA-Cy5 (red) (4 h at 37 °C). Cell nuclei were counterstained with Hoechst (blue). Scale bars represent 25 μm. (**B**) FACS results showing controls of untreated control cells (Q4, blue), cells positive for Cy5 (treated with SNs-ST-Cy5, Q1, red), cells positive for TopFluor^®^ (treated with SNs-ST-TopFluor^®^, Q3, green), and double positive cells for Cy5 and TopFluor^®^ (treated with either SNs-ST-Cy5-TopFluor^®^, Q2, orange, or SNs-Lpx-Cy5-TopFluor^®^, Q2, pink). (**C**) FACS Histogram of two different fluorophores, TopFluor^®^ and Cy5, obtained for cells treated with double labeled nanoparticles, (SNs-ST-Cy5-TopFluor^®^, orange, or SNs-Lpx-Cy5-TopFluor^®^, pink).

We next proved, making use of confocal microscopy and flow cytometry, that both formulations were efficiently internalized by cancer cells (green signal corresponding to the SM-TopFluor^®^ of SNs-ST and SNs-Lpx) and mediated an efficient intracellular delivery of the associated miRNA (labeled with Cy5), which was observed into the cell cytoplasm (red signal). Importantly, merged images evidenced that in both cases, SNs-ST and SNs-Lpx were taken up by the cells as an assembled form of stable nanosystems, in view of the good co-localization of the green and red signals in both cases (Figure 3A). SNs-ST and SNs-Lpx showed the potential to deliver oncosuppressor miRNAs to cancer cells. The surface charge of nanocarriers plays an important role in their cellular uptake and internalization pathway. Cationic nanocarriers can bind to sulfated proteoglycan of cell surfaces through electrostatic interactions and subsequently be internalized by pinocytosis and endocytosis [43]. The fact of using two fluorescent dyes allowed us confirming that both labeled molecules were traveling together forming a nanostructure. This approach has already been reported by others, as for example, the co-delivery of doxorubicin and Bmi1 siRNA by folate receptor-targeted liposomes and the doxorubicin-loaded fluorescent nanovehicle [44,45].

Complementary, FACS analysis was used to verify the results and provide quantitative data (Figure 3B,C). Three controls were established in the FACS experiment from the fluorescent dyes (Cy5 and TopFluor^®^), allowing us identifying (i) cells that do not receive any treatment (double-negative control cells, Cy5 −, TopFluor^®^ −), located in quadrant 4 (Q4), (ii) cells positive for Cy5 (Cy5 +, TopFluor^®^ −), located in quadrant 1 (Q1) and (iii) cells positive for TopFluor^®^ (Cy5 −, TopFluor^®^ +) located in quadrant 3 (Q3). Apart from these tree controls, double-positive cells (Cy5 +, TopFluor^®^ +) treated with either SNs-ST or SNs-Lpx are located in quadrant 2 (Q2). Remarkably, these double-positive cells corroborate that the nanoparticles labeled with SM-TopFluor^®^ effectively delivered miRNA-Cy5 into the cells, and confirms that both molecules are actually traveling together into the nanostructure. Importantly, SNs-Lpx showed a higher internalization percentage for miRNA-Cy5 (92% of double-positive cells for SNs-Lpx and 57% for SNs-ST), while the TopFluor^®^ signal was comparable for both formulations (Figure 3C). This could be associated with a higher miRNA protection when the nucleic acids are encapsulated into SNs in the form of Lpx, avoiding displacement and premature release. This fact has been previously reported for nanosystems in which nucleic acids are attached onto the nanoparticle surface by establishing electrostatic interactions [46]. Moreover, these cationic Lpx might be able to destabilize the cell membranes by themselves and penetrate into the cells even if dissociated from the nanoparticles [47,48].

### 3.3. Transfection Efficiency

**Figure 4:**
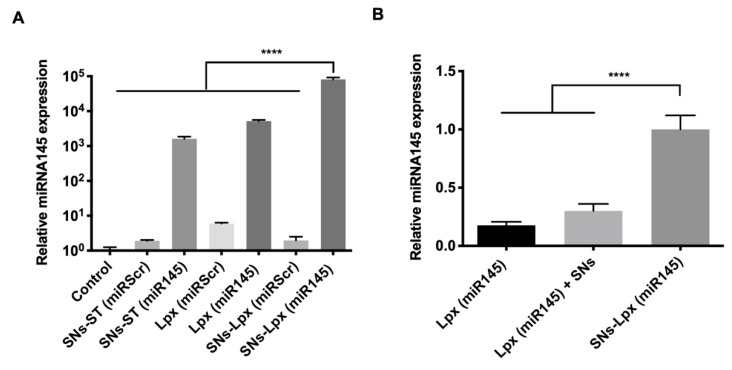
(**A**) Expression of miRNA145 after transfection with SNs-ST (miR145), Lpx (miR145), and SNs-Lpx (miR145) nanosystems (as well as nanosystems prepared with the scrambled sequence, SNs-ST (miRScr), Lpx (miRScr), and SNs-Lpx (miRScr). (**B**) Relative expression of miR145 after transfection with Lpx (miR145), a physical mixture of Lpx (miR145) and SNs, and SNs-Lpx (miR145) in SW480 cells (2 μg miRNA/well in 6-well plates, 4 h incubation, 72 h post-transfection, data normalized against RNU6) (**** *p* < 0.0001).

SW480 cells were transfected with SNs-ST and SNs-Lpx loaded with miRNA145 (miR145) and with the same formulations but with a scrambled sequence for control (miRScr). The transfected cells were analyzed by qRT-PCR. (Figure 4A). Results showed that all formulations loaded with miR145 were able to efficiently increase the intracellular levels of this oncosuppressor miRNA compared to control cells. Interestingly, SNs-Lpx (miR145) showed a significantly higher transfection efficiency in relation to SNs-ST (miR145) (64-fold increase), in agreement with the cell internalization studies reported in the previous section, showing a higher cell internalization for miRNA-Cy5 for this formulation, and with the agarose gels that revealed and improved stability of the encapsulated miRNA. The preformed Lpx later encapsulated in SNs-Lpx, provided a supportive protection for miRNA145 and mediated an improved its delivery. Transfection efficiency experiments with Lpx and a physical mixture of Lpx with SNs (Lpx + SNs) were additionally carried to determine if the positive effect of SNs-Lpx could be simply attributed to the fact that Lpx were present in the formulation. Results evidenced again the superior performance of the formulation that encapsulates Lpx (SNs-Lpx), probably due to a protective effect of the miRNA145 (Figure 4B). Importantly, results obtained with SNs-Lpx were superior to those recently published with miRNA145-loaded PLGA/PEI/HA nanoparticles in HTC-116 cells (11-fold increase), miRNA-145-loaded magnetic nanoparticles in AsPC-1cells (19-fold increase) and HPAF-II cells (4-fold increase), miRNA145-loaded cationic liposomes in HepG2 cells (9-fold increase), miRNA145 associated to a lentiviral vector in SW620 cells (8.2-fold increase), and miRNA145-loaded protamine nanocapsules in SW480 cells (33-fold increase) [15,16,17,49,50]. In relation to nanometric porous metal-organic frameworks (nanoMOFs), they show a similar efficiency in SW480 cells, superior to lipofectamine, as recently published by our group [51].

### 3.4. Anticancer Activity

**Figure 5:**
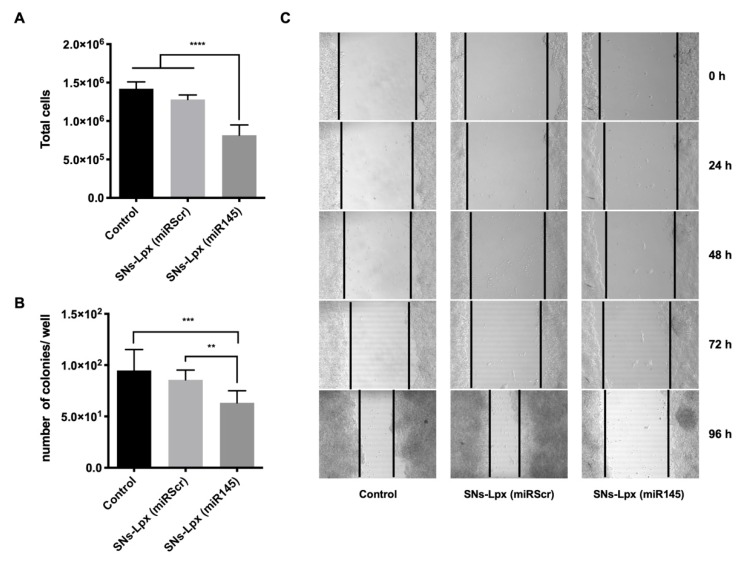
(**A**) Anticancer activity of SNs-Lpx (miR145) in SW480 transfected cells. Proliferation of SW480 cells 72 h post-transfection (**** *p* < 0.0001). (**B**) Quantitative analysis of colony numbers, 2 weeks after transfection is shown (*** *p* < 0.001 and ** *p* < 0.01). (**C**) Representative images showing cell migration in wound healing assay, of untreated control cells and cells treated with SNs-Lpx (miR145) and the same formulation with the scrambled sequence (SNs-Lpx (miRScr)). Images (magnification 10x) were taken 0, 24, 48, 72, and 96 h after treatment. The dark lines designated the rough margins of the SW480 cells.

Experiments were finally conducted to determine the therapeutic potential of the selected formulation SNs-Lpx to interfere with the tumorigenic capacity of SW480 colorectal cancer cells. As shown in Figure 5A, cells treated with the formulation that associate the oncosuppressor miRNA145, proliferate less than the control cells and cells treated with the scrambled sequence. On the other hand, cells transfected with SNs-Lpx (miR145) also have a lower capacity for colony formation with respect to control cells and cells transfected with the scrambled miRNA (Figure 5B). Results from a wound healing assay, one of the more referred to techniques for cell migration in cancer research, also highlight the good performance of SNs-Lpx (miR145). Indeed, the artificially created wound of SW480 treated with SNs-Lpx (miR145) remained almost inalterable at the end of the study, oppositely to what was observed in the case of untreated cells and cells treated with the scrambled sequence SNs-Lpx (miRScr) (the wound closure was superior to 50%) (Figure 5C). Quantitative analysis shows significant differences at 96 h (Figure S6). Interference with these tumor cell properties, proliferation, migration, and capacity of colony forming, is well related to a decreased tumorigenic capacity [52,53]. According to this, our results clearly indicated that SNs-Lpx that associated oncosuppressor miRNA145, inhibited cell growth and migration in colorectal cancer cells and highlighted the potential of this formulation for avoiding tumor progression in colorectal cancer.

## 4. Conclusions

We have successfully proved the potential of SNs for the efficient association of miRNA145 mimics to interfere with cancer progression in colorectal cancer. We pursued two different strategies to associate miRNA, i.e., adsorption to the surface of cationic SNs, SNs-ST (miR), and preformation of complexes for further encapsulation into SNs, SNs-Lpx. Both formulations showed the capacity to deliver miRNA145 intracellularly and, thereafter, efficiently restore its expression. Restitution of miRNA145 levels from the delivery of SNs-Lpx demonstrated to be related to the strongest anticancer effect, in view of the efficiency shown in tumor proliferation, colony formation, and migration capacity assays. Therefore, we can conclude that this formulation holds a strong potential for the development of efficient miRNA replacement therapies for the treatment of colorectal cancer.

## Figures and Tables

**Table 1:** Physicochemical properties of SNs-ST.

Formulation	^a^ ST (*w*/*w*)	Size (nm)	^b^ PDI	ζ-Potential (mV)
	-	131 ± 8	0.2	−13 ± 7
SNs-ST	0.1	104 ± 10	0.2	+40 ± 12
	0.5	115 ± 11	0.2	+44 ± 4
	1	109 ± 11	0.2	+46 ± 6
	1.5	99 ± 12	0.3	+48 ± 11
	2	104 ± 22	0.3	+51 ± 3

Data presented as mean ± standard deviation (*n* = 3). **^a^** The *w*/*w* ratio of ST refers to the amount of VitE in the formulation (SNs were prepared in all cases at a constant. VitE:SM:PEG ratio of 10:1:0.1). ^b^ PDI: polydispersity index.

**Table 2:** Physicochemical properties of SNs-ST (miR).

Formulations	Mass Ratio (*w*/*w*) ^a^ miRNA:ST	Theoretical miRNA Loading (%)	Size (nm)	PDI ^b^	ζ-Potential (mV)
SNs-ST (miR)	1:10	0.8	158 ± 8	0.2	+26 ± 3
	1:7.5	1.1	Precipitate		
	1:5	1.6	Precipitate		
	1:2.5	3.2	172 ± 4	0.2	−15 ± 2
	1:1	7.7	155 ± 2	0.2	−30 ± 3

Data presented as mean ± standard deviation (*n* = 3), ^a^ miRNA was maintained constant (10 μg per formulation), and ^b^ PDI: polydispersity index.

## Supplementary Materials

The following are available online at https://www.mdpi.com/1999-4923/12/2/189/s1, Table S1. Physicochemical properties of Lipid complexes (miRNA-ST and miRNA-DOTAP); Figure S1: Characterization of SNs. (**A**) Size distribution graph measured from nanoparticle tracking analysis (NTA) and dynamic light scattering (DLS) and (**B**) video frame acquired by NTA. Figure S2. Electrophoresis of SNs-ST (miR) in different ratios of miRNA:ST (**A**) (1:10 and 1:2.5) and (**B**) (1:1) with or without heparin, control miRNA 0.5 μg (agarose 1% 100 V, 40 min). Figure S3: Characterization of SNs-ST and SNs-ST (miR). (**A**) Transmission electron microscopy (TEM) images. (**B**) Size distribution graph measured from nanoparticle tracking analysis (NTA) and dynamic light scattering (DLS), and (**C**) video frame acquired by NTA. Figure S4: Electrophoresis of Lpx and SNs-Lpx. A displacement experiment upon incubation with a 25-fold excess of heparin (*w*/*w*) for 2 h at 37 °C allowed the migration of the associated miRNA. Experiment was carried in agarose gel 1% 100 Volts, 40 min (control miRNA 0.5 μg). Figure S5. Cellular viability of SW480 cells after incubation with increased concentrations of SNs-ST and SNs-Lpx (24 h at 37 °C). Figure S6. Normalized wound closure (%) after treatment of SW480 cells with SNs-Lpx (miR145), control, and the formulation with the scrambled sequence (SNs-Lpx (miRScr)).
