# Supplementary material for: Sphingomyelin-Based Nanosystems (SNs) for the Development of Anticancer miRNA Therapeutics
Source: Pharmaceutics. 2020 Feb 22;12(2):189. doi: 10.3390/pharmaceutics12020189 (PMC7076701; doi:10.3390/pharmaceutics12020189)
Supplement: Supplementary file 1 [file pharmaceutics-12-00189-s001.pdf]

pharmaceutics

Supplementary Materials: Sphingomyelin-Based Nanosystems (SNs) for the Development of Anticancer miRNA Therapeutics

Surasa Nagachinta, Belen Lopez Bouzo, Abi Judit Vázquez-Ríos and **Rafael López and María de la Fuente**

Table S1. Physicochemical properties of Lipid complexes (miRNA-ST and miRNA-DOTAP).

Lipid Complexes	Mass ratio (*w*/*w*) [a] miRNA:Cationic Lipid	Size (nm)	PDI [b]	ζ-Potential (mV)
miRNA-ST	1:1	466± 91	0.4	−9 ± 4
	1:5	76 ± 86	0.7	+17 ± 4
	1:10	158 ± 96	0.6	+28 ± 6
	1:15	189 ± 54	0.3	+29± 4
	1:20	164 ± 45	0.3	+31 ± 3
miRNA-DOTAP	1:1	15 ± 18	0.7	−10 ± 4
	1:5	68 ± 73	0.7	+6 ± 7
	1:10	46 ± 34	0.4	+20 ± 7
	1:15	83 ± 16	0.3	+32 ± 3
	1:20	123 ± 80	0.5	+32 ± 3

Data presented as mean ± standard deviation (n = 3), [a] miRNA was maintained constant (10 µg per formulation), [b] PDI: polydispersity index.

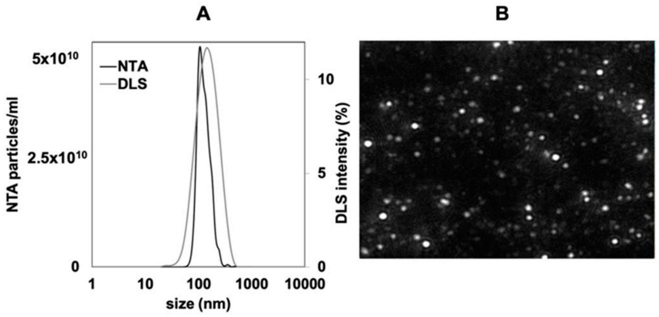

Figure S1. Characterization of SNs. (**A**) Size distribution graph measured from nanoparticle tracking analysis (NTA) and dynamic light scattering (DLS) and (**B**) video frame acquired by NTA.

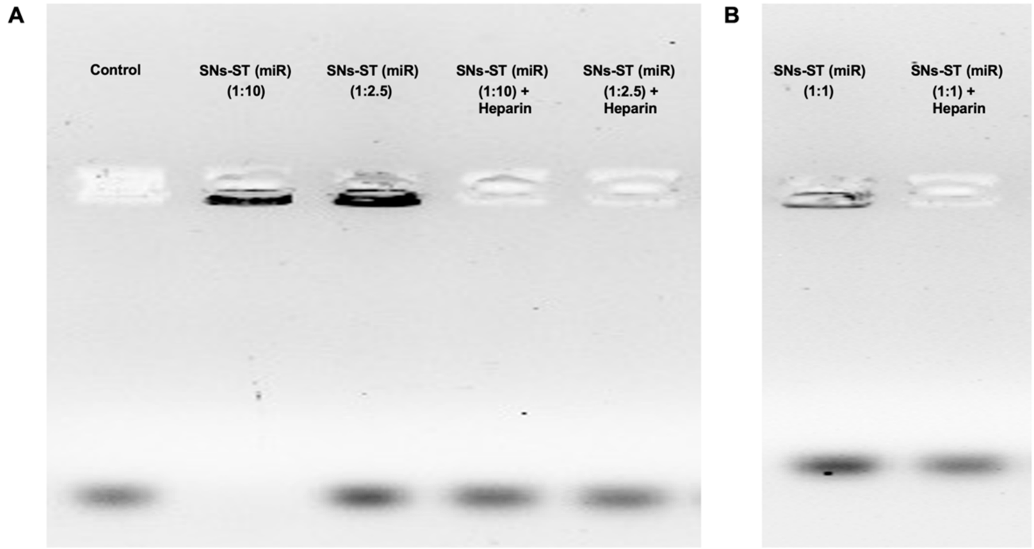

Figure S2. Electrophoresis of SNs-ST (miR) in different ratios of miRNA:ST (**A**) (1:10 and 1:2.5) and (**B**) (1:1) with or without heparin, control miRNA 0.5 μg (agrarose 1% 100 V, 40 min).

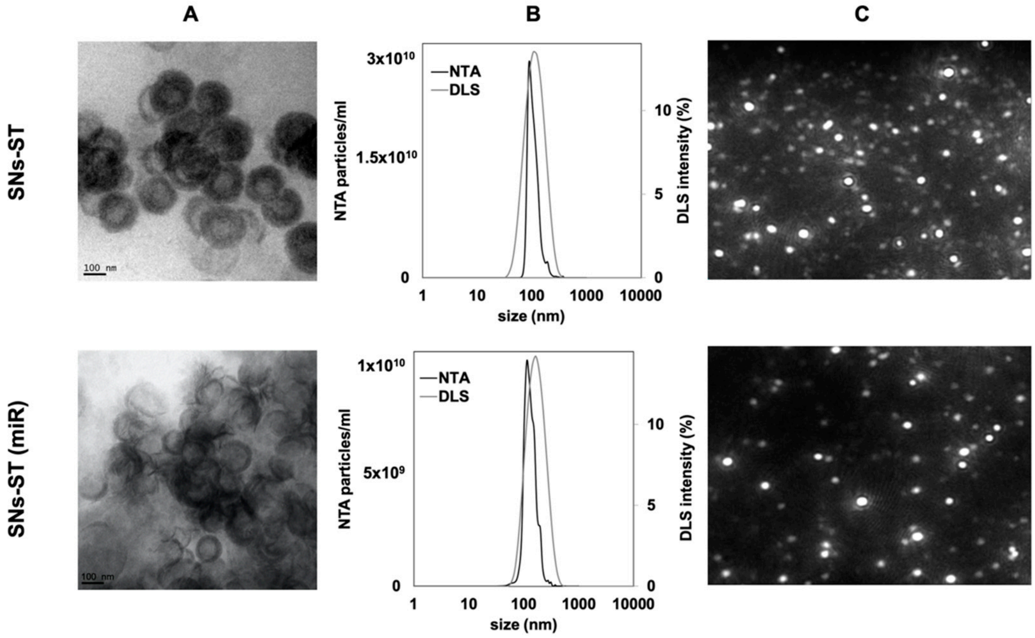

Figure S3. Characterization of SNs-ST and SNs-ST (miR). (**A**) Transmission electron microscopy (TEM) images. (**B**) Size distribution graph measured from nanoparticle tracking analysis (NTA) and dynamic light scattering (DLS), and (**C**) video frame acquired by NTA.

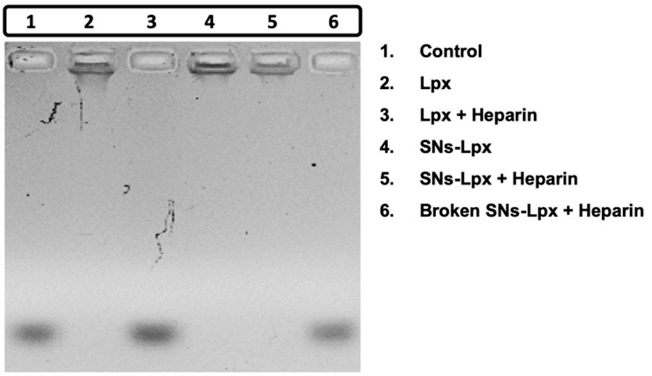

Figure S4. Electrophoresis of Lpx and SNs-Lpx. A displacement experiment upon incubation with a 25-fold excess of heparin (*w*/*w*) for 2 h at 37 °C allowed the migration of the associated miRNA. Experiment was carried in agarose gel 1% 100 Volts, 40 min (control miRNA 0.5 μg).

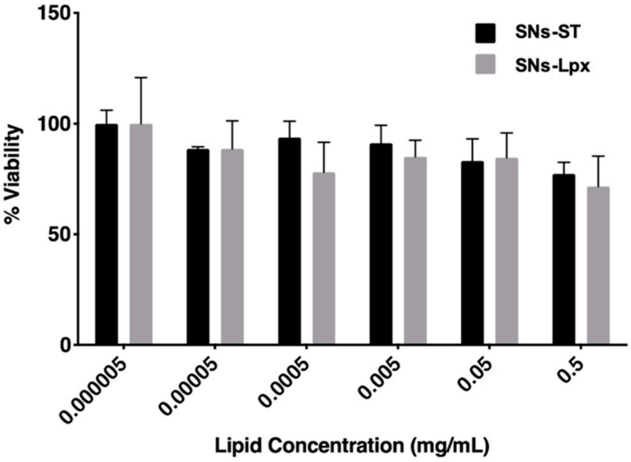

Figure S5. Cellular viability of SW480 cells after incubation with increased concentrations of SNs-ST and SNs-Lpx (24 h at 37 °C).

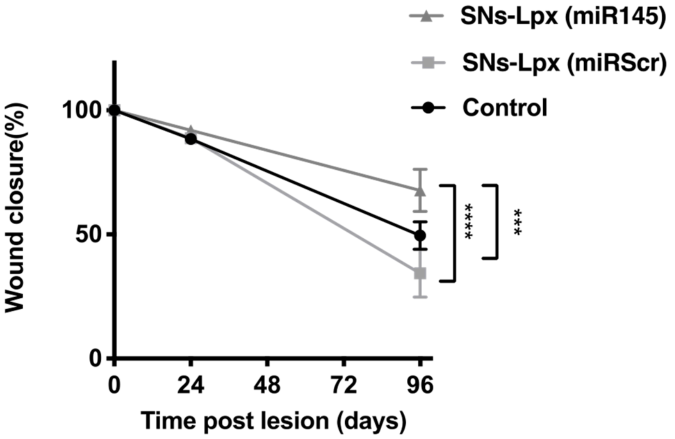

Figure S6. Normalized wound closure (%) after treatment of SW480 cells with SNs-Lpx (miR145), control, and the formulation with the scrambled sequence (SNs-Lpx (miRScr)).
